# “It's Okay, Everyone Else Is Doing It”: Moral Disengagement and Peer Delinquency

**DOI:** 10.1002/jad.70203

**Published:** 2026-06-15

**Authors:** Romain Decrop, Michael McCart

**Affiliations:** ^1^ Department of Psychology Bowling Green State University Bowling Green Ohio USA; ^2^ Department of Psychiatry and Behavioral Sciences Medical University of South Carolina Charleston South Carolina USA

**Keywords:** adolescent, development, moral disengagement, peer delinquency, young adult

## Abstract

**Introduction:**

Adolescence is a developmental period during which moral cognition and peer environments change in ways that shape trajectories of antisocial behavior into adulthood. Although moral disengagement (MD) and peer delinquency (PD) are established risk factors for persistent offending, they are typically studied in isolation. The present study investigates the joint development of MD and PD across adolescence and early adulthood, and tests whether changes in one process forecast subsequent changes in the other.

**Methods:**

Self‐reported survey data of MD and PD from 1,354 U.S. justice‐involved youth (86.41% male; baseline Mage = 16.04; 41.5% Black, 33.5% Hispanic, 20.2% White, 4.8% Other) in the Pathways to Desistance study were analyzed using an autoregressive latent trajectory framework across 11 waves that spanned 7 years from 2000 to 2010.

**Results:**

MD and PD exhibited coordinated long‐term declines with aligned slowing over time, such that higher levels in either process were linked to more persistent elevations in the other. At the within‐person level, autoregressive continuity varied across development, with stronger short‐term persistence during the middle waves and attenuation at earlier and later stages. Similarly, cross‐lagged associations were small and wave‐specific, emerging only at selected transitions rather than reflecting consistent reciprocal prediction across waves.

**Conclusions:**

Together, these findings demonstrate that moral cognition and peer contexts are dynamically linked across adolescence and adulthood, highlighting the potential value of considering both processes in future work aimed at understanding persistent antisocial trajectories.

## 1 Introduction

Adolescence is marked by cognitive and social changes that heighten risk‐taking and shape offending trajectories into adulthood (Moffitt 1993; Steinberg 2008). During this period, youths' emerging capacity for abstract reasoning and identity formation coincides with expanding social networks, making both moral cognition and peer environments central to antisocial behavior. Within this context, moral disengagement (MD) refers to the cognitive mechanisms that justify, minimize, or reframe harmful behavior (Bandura et al. 1996). At the social level, peer delinquency (PD) captures the extent to which youths' friends engage in antisocial behavior, creating contexts that can model, reinforce, and normalize misconduct (Dishion & Tipsord 2011). Although MD and PD are established risk factors for persistent offending, their joint development remains understudied (Paciello et al. 2008; Thornberry et al. 1994). Accordingly, clarifying how moral cognition and peer contexts become linked over time may reveal developmentally targeted opportunities to disrupt offending trajectories.

### 1.1 Moral Disengagement

Moral theories suggest that individuals avoid behaviors that trigger negative self‐appraisals because moral violations can evoke aversive emotions (e.g., shame, guilt; Festinger 1957). To circumvent these, some youth rely on MD to weaken self‐sanctions and permit norm violations (Bandura et al. 1996). First, they may “talk themselves into” antisocial acts through moral justifications, euphemistic language, or advantageous comparisons (e.g., “All my friends steal from that store,” “That's how it is with my friends, it's no big deal,” “I've seen my friends do worse”). They may also downplay harm or shift responsibility to peer pressure, shared decision‐making, or group expectations (e.g., “It wasn't my idea, they made me do it,” “Everyone was doing it”). If harm becomes too salient, youth may cope by blaming or dehumanizing the victim (e.g., “They deserved it for messing with my friend,” “They're not one of us”). Through these mechanisms, moral self‐regulation is temporarily disengaged, making antisocial acts feel permissible by decreasing perceived costs and increasing anticipated rewards (Decrop et al. 2025). Accordingly, MD has been linked to greater aggression, delinquency, and substance use through adolescence (Decrop et al. 2026; Gini et al. 2014).

Longitudinally, MD tends to rise from early to mid‐adolescence, as growing perspective‐taking and abstract reasoning allow youth to reinterpret harmful behaviors, before declining into early adulthood as self‐regulation, accountability, and internalized moral standards strengthen (Paciello et al. 2008; Shulman et al. 2011). This peak aligns with Kohlberg's conventional stage, when moral judgments are shaped by social approval and conformity, increasing motivation to justify behaviors that conflict with internalized values (Kohlberg 1981). Neurodevelopmentally, heightened MD likely reflects underdeveloped prefrontal systems supporting cognitive control, perspective‐taking, and moral reasoning, alongside weaker integration with limbic regions involved in empathy and emotional processing (Decety & Cowell 2014; Galvan et al. 2007). As these systems mature into adulthood, moral reasoning becomes more affectively integrated and reliance on disengagement declines (Hyde et al. 2010; Moore et al. 2012). Notably, MD declines from adolescence to adulthood are associated with offending reductions among justice‐involved youth (Caprara et al. 2014; Shulman et al. 2011). However, since adolescent rule‐breaking often occurs within peer contexts, MD's developmental significance likely depends on the social environments in which the cognitive processes unfold (Dishion et al. 1996; Warr 2002).

### 1.2 Peer Delinquency

Like MD, antisocial peer affiliation is among the strongest predictors of delinquency (Dishion & Tipsord 2011; Pardini et al. 2005). This association reflects social mechanisms that model rule‐breaking, reinforce deviant talk and actions, and normalize and reward offending (Dishion et al. 1996; Warr 2002). Developmentally, PD rises during early to mid‐adolescence as peer‐status sensitivity, time with friends, and risk‐taking increase while adult supervision decreases (Steinberg & Monahan 2007; Warr 2002). Then, by late adolescence, PD declines as social networks diversify, autonomy increases, and peers desist from offending, reducing both the availability and influence of delinquent groups (Moffitt 1993; Warr 2002). Peer selection and socialization processes help explain this developmental course, reinforcing antisocial behavior when networks are stable but weakening them as social contexts reorganize. As such, delinquent peer affiliation is best understood within a dynamic social ecology, raising the question of how adolescents' moral reasoning and peer contexts shape one another over time.

### 1.3 Moral Disengagement and Peer Delinquency

As adolescents gain autonomy and spend more time with peers, their moral beliefs and social environments increasingly align during identity formation through two complementary processes: selection into peer groups that reflect one's tendencies and socialization within those groups (Patterson et al. 1989; Thornberry & Krohn 2005). Interactional models propose that early cognitive vulnerabilities and conduct problems weaken bonds to prosocial contexts and increase opportunities for delinquent peer involvement (Thornberry & Krohn 2005). From this perspective, youth who use more MD may be more comfortable with rule‐breaking and justification‐based reasoning and less constrained by guilt, lowering barriers to affiliating with delinquent peers who hold similar views or also use MD. Yet, empirical tests of MD as an antecedent of PD are scarce, with longitudinal studies predominantly examining MD as a predictor of behaviors rather than peer networks. Thus, it remains unclear whether, and when, disengaged moral reasoning guides selection into deviant peer contexts.

Alternatively, socialization models suggest that delinquent peer affiliations expose adolescents to rule‐breaking discussions, antisocial modeling, and reinforcement of norm‐violations that shape moral standards through observational learning, social persuasion, and exposure to norm‐breaking by valued others (Bandura 1991; Dishion et al. 1996; Warr 2002). Consequently, in groups where offending is excused, victims are derogated, and harm is justified or minimized, youth have repeated opportunities to practice MD with peer affirmation. Over time, these environments may increase MD, or slow its normative decline, by normalizing disengaged interpretations of harm. Indeed, exposure to peers who are permissive of norm‐breaking, model or reinforce antisocial behavior, or tacitly condone harm has been linked to greater adoption of disengaged moral reasoning into adulthood (Gini et al. 2014; Pozzoli et al. 2016; Shulman et al. 2011). Although these studies emphasize peer norms rather than behavior, they suggest that harm‐tolerating environments contribute to increases in MD. However, the limited focus on peer behavior remains a gap in the literature, as behavioral exposure provides the most direct opportunities for modeling and reinforcement.

Over time, these exposures may become embedded in mutually reinforcing selection and socialization processes, allowing youth and peers to jointly escalate externalizing problems. Empirically, early antisocial behaviors predict later delinquent peer affiliations, which forecast subsequent problem behaviors (Pardini et al. 2005; Thornberry & Krohn 2005). While this reciprocal pattern suggests that adolescents and their peer environments continually shape one another, no work has tested whether MD operates within this transactional developmental system. Through this lens, MD and PD may become linked through self‐reinforcing selection and socialization processes, as disengaged moral reasoning shapes youths' peer contexts and those contexts, in turn, alter moral reasoning. Clarifying this dynamic is critical for identifying whether and when targeting peer contexts, moral cognition, or both may best alter risk trajectories.

### 1.4 Current Study

This study examined whether MD and PD co‐develop as components of a transactional developmental system among justice‐involved youth across adolescence and early adulthood. Rather than treating disengaged moral cognition and delinquent peer contexts as isolated predictors of antisocial behavior, we tested whether MD may shape youths' selection into, and maintenance of, delinquent peer contexts while PD may reinforce disengaged moral reasoning. Moreover, prior work on the topic has relied on models that obscure stable between‐person differences (e.g., youth with consistently high levels), coordinated developmental change (e.g., parallel declines), and time‐specific reciprocal influence (e.g., elevations in one process predicting change in the other). Using an autoregressive latent trajectory (ALT) framework, we modeled long‐term between‐person trajectories and within‐person deviations capturing short‐term persistence and reciprocal coupling. Justice‐involved youth provide a useful context for these dynamics because they often show elevated exposure to delinquent peers, morally disengaged reasoning, and heterogeneity in developmental risk trajectories, while also experiencing conditions that shape peer access (e.g., residential placement, supervision, reentry; Shulman et al. 2011; Warr 2002). We hypothesized that MD and PD would exhibit coordinated developmental trajectories, autoregressive continuity, and wave‐specific cross‐lagged effects from adolescence to adulthood.

## 2 Materials and Methods

### 2.1 Participants

Data were from the Pathways to Desistance study, an investigation of 1354 juvenile offenders from Philadelphia, PA (*n* = 700) and Phoenix, AZ (*n* = 654). At baseline, participants were 14–17 years old (*M* = 16.04, SD = 1.14), predominantly male (*n* = 1170, 86.41%), charged with a felony or serious nonfelony, and identified as: 41.5% Black, 33.5% Hispanic, 20.2% White, and 4.8% another race/ethnicity.

### 2.2 Procedures

Researchers recruited 2008 youth, 67% of whom completed baseline interviews and were reinterviewed every 6 months for 3 years and then annually for 4 years (i.e., 11 timepoints across 7 years). Data collection spanned from November 2000 to March 2010 with high retention rates (range = 84%–94%, M = 90%; male range = 1170–962; female range = 184–172; Schubert et al. 2004). The original study was approved by the Institutional Review Boards (IRB) of principal investigators' universities, and parental and/or youth consent permitted future secondary analyses. The present study analyzed publicly available de‐identified data (icpsr.umich.edu/web/NAHDAP/studies/29961) and did not require Bowling Green State University's IRB approval. Additional study details are described at pathwaysstudy.pitt.edu.

### 2.3 Measures

Descriptive information, Cronbach's alphas, rank‐order stability (i.e., consecutive wave correlations), and Pearson correlation coefficients are in Table A1 and Supplementary Table 1. MD and PD measure items are in Supplemental Tables 2 and 3.

### 2.4 Moral Disengagement

MD was assessed at each wave with the Mechanisms of Moral Disengagement scale (Bandura et al. 1996), which consists of 32 3‐point Likert scale items (1 “Disagree,” 2 “Neither agree nor disagree,” 3 “Agree”; e.g., “Kids cannot be blamed for misbehaving if their friends pressured them to do it.”). Similar to Decrop et al. (2025), we used a total mean score with higher values indicating a more permissive attitude towards morally disengaging.

### 2.5 Peer Delinquency

We measured PD at each wave using 12 5‐point Likert scale items adapted from the Rochester Youth Study (Thornberry et al. 1994) that asked participants how many of their friends engaged in antisocial behaviors (e.g., selling drugs, robbery; 1 “None of them”, 5 “All of them”). Baseline items asked about the past 6 months and follow‐up waves since the last recall. A total mean score was used with higher scores indicating a greater number of delinquent peers.

### 2.6 Analytical Plan

Models were estimated in Mplus 8.8 using a robust maximum likelihood estimator. Missing data were handled with full‐information maximum likelihood, which retained all cases with available data under a missing‐at‐random assumption based on observed model variables (Muthén & Muthén 1998–2017). Attrition checks indicated that baseline MD and PD did not significantly predict final‐wave missingness or the number of completed waves, either before or after adjusting for age, sex, and race/ethnicity. Observed scale means, rather than latent factors, were used to reduce ALT model complexity and improve convergence, though unmodeled measurement error may have biased associations. Nevertheless, we identified the optimal unconditional growth forms of MD and PD by estimating separate 11‐wave latent growth models comparing intercept‐only, linear, and quadratic specifications, with growth factors allowed to vary across individuals to capture differences in initial levels and change (Curran & Hussong 2003). Intercept loadings were fixed at 1, linear loadings were fixed to 0, 0.1, 0.2, 0.3, 0.4, 0.5, 0.6, 0.8, 1.0, 1.2, and 1.4 to reflect the 6‐month (Waves 1‐7) and 12‐month spacing (Waves 8–11), and quadratic loadings were squared linear values (0 to 1.96). Model selection favored lower AIC, BIC, SABIC, RMSEA, and SRMR, higher CFI and TLI, and significant chi‐square difference tests for nested models.

**Figure A1:**
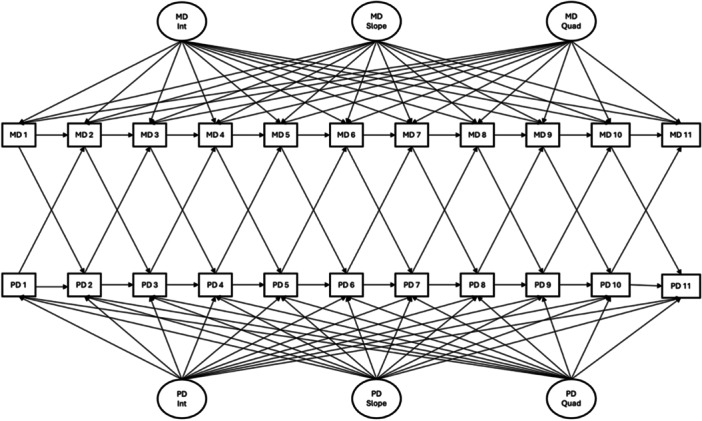
*Autoregressive Latent Trajectory Model. Note*. Int = intercept, Slope = linear growth factor, Quad = quadratic growth factor, MD# = moral disengagement at Wave #, PD# = peer delinquency at Wave #. The figure depicts the autoregressive latent trajectory model. Growth factors capture between‐person differences in initial levels and change, whereas autoregressive and cross‐lagged paths capture within‐person carryover and reciprocal coupling across adjacent waves. Correlations among growth factors and residuals are omitted for clarity.

The best‐fitting models were then embedded within an ALT framework using the same time‐scaled growth loadings to examine the joint MD‐PD development across waves (Bollen & Zimmer 2010; Figure A1). ALT models integrate latent growth and cross‐lagged panel approaches by estimating long‐term trajectories alongside wave‐specific deviations. Thus, in addition to covariances among growth factors within and across constructs, ALT models also include autoregressive paths within each construct, cross‐lagged paths between MD and PD across successive waves, and correlated residuals for within‐wave associations. This specification separates stable between‐person differences in overall levels from within‐person, time‐specific fluctuations that test whether deviations in one construct predict deviations in the other.

After estimating an unconstrained ALT model (Model 1), we tested nested constraints on autoregressive and cross‐lagged associations. Model 2 constrained autoregressive paths to equality across waves separately for MD‐ > MD and PD‐ > PD, testing time‐invariant within‐construct carryover. Model 3 additionally constrained MD‐ > PD and PD‐ > MD cross‐lagged paths, imposing full temporal invariance. Model 4 retained Model 2's autoregressive constraints but allowed cross‐lagged effects to differ between Waves 1–7 (6‐month spacing) and 8–11 (annual spacing), testing whether cross‐lagged MD‐PD associations differed across assessment intervals. Models 5 and 6 also constrained autoregressive paths like Model 2 but tested asymmetric structures by constraining cross‐lagged paths in only one direction (i.e., MD‐ > PD in Model 5; PD‐ > MD in Model 6), while freely estimating the reciprocal direction. Model fit was evaluated using lower AIC, BIC, SABIC, RMSEA, and SRMR and higher CFI and TLI.

## 3 Results

MD and PD were correlated within and across waves (all *p*s < 0.01; Supplemental Table 1), indicating sustained coupling and support for an ALT framework.

### 3.1 Unconditional Growth Curve Models

The unconditional latent growth models revealed that quadratic growth best fit both constructs (Table A2 & Supplemental Table 3). On average, MD and PD scores began at 1.60 and 2.13, respectively, and declined over time, with decreases decelerating or plateauing as most youth reached young adulthood (all growth factor *p*s < 0.001). Covariances suggested regression‐to‐the‐mean effects, where youth with higher baseline MD or PD showed steeper declines in their respective construct, which were associated with greater deceleration and leveling off. Significant intercept and slope variances also indicated heterogeneity in initial levels and rates of change (all *p*s < 0.001), such that youth differed in their starting levels and developmental trajectories. However, since these models did not account for concurrent change in the other construct, we next estimated ALT models with quadratic growth curves.

### 3.2 Autoregressive Latent Trajectory Model

The fully unconstrained model (Model 1) fit best (Supplemental Table 4; AIC = 30461.38, BIC = 30982.47, SABIC = 30664.81, RMSEA = 0.014, CFI = 0.994, TLI = 0.992, SRMR = 0.036). Constraining autoregressive paths (Model 2) generally reduced fit (AIC = 30517.83, BIC = 30945.12, SABIC = 30684.64, RMSEA = 0.019, CFI = 0.988, TLI = 0.986, SRMR = 0.040), as did additional cross‐lagged constraints, whether full (Model 3), developmentally segmented (Model 4), or direction‐specific (Models 5 & 6; Models 3‐6 ranges: AIC = 30585.45–30737.96; BIC = 30965.84–31079.29; SABIC = 30733.95–30868.15; RMSEA = 0.024–0.031; CFI = 0.966–0.981; TLI = 0.962–0.978; SRMR = 0.041–0.044). These comparisons supported the unconstrained model, indicating that time‐invariant autoregressive or cross‐lagged constraints did not capture the data as adequately. Retaining the unconstrained specification therefore allowed MD‐PD coupling to vary in strength and direction across assessment transitions, consistent with the study's focus on wave‐specific developmental patterning. However, since autoregressive and cross‐lagged paths represent adjacent‐assessment associations rather than effects scaled to a common lag duration, wave‐specific differences may reflect both maturation and assessment spacing. Still, as described below, significant effects appeared across both six‐ and twelve‐month intervals, suggesting that the observed patterns were not confined to one interval length. With this in mind, our interpretations focus on the unconstrained model.

### 3.3 Latent Growth Curve Component

Table A3 reveals moderate‐to‐large associations between MD and PD trajectories, indicating substantial long‐term coupling. Higher initial MD corresponded to higher initial PD (intercept‐intercept latent growth factors), steeper MD declines to steeper PD declines (slope‐slope), and greater MD deceleration to greater PD deceleration (quadratic‐quadratic; std est range = 0.48–0.60, *p*s < 0.001). Beyond these parallel processes, higher initial MD predicted slower PD declines (intercept–slope) and greater PD deceleration (intercept–quadratic; std est = −0.39 and 0.36, respectively, ps < 0.001), while higher initial PD predicted slower MD declines and greater MD deceleration (std est = −0.54 and 0.53, respectively, *p*s < 0.001). Cross‐construct slope–quadratic associations were also significant, with steeper MD and PD declines linked to greater deceleration in the other construct (std est = −0.43 and −0.47, respectively, *p*s < 0.001).

### 3.4 Autoregressive Component

**Figure A2:**
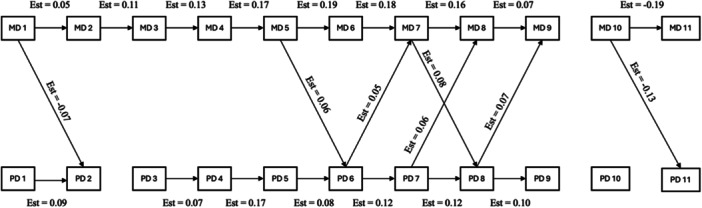
*Significant Autoregressive and Cross‐lagged Components in the ALT Model. Note*. N = 1354; MD# = moral disengagement at Wave #, PD# = peer delinquency at Wave #. The figure depicts the significant autoregressive and cross‐lagged components of the autoregressive latent trajectory model (i.e., within‐person carryover and reciprocal coupling across adjacent waves, respectively). Correlations among growth factors and residuals are omitted for clarity.

At the within‐person level, both MD and PD showed significant autoregressive continuity across most waves, with temporary increases above individuals' developmental trajectories often persisting to the next wave (Table A4 & Figure A2). For MD, carryover was weak early (Waves 1–2; *M*_ages_ ≈ 16–17), strengthened during middle waves (Waves 3–7; *M*_ages_ ≈ 17–19), briefly attenuated (Waves 8–9; *M*_ages_ ≈ 20–21), and then became nonsignificant by the final transition, where the association reversed (Waves 10–11; *M*_ages_ ≈ 22–23). Similarly, PD exhibited modest early effects (Waves 1–2; *M*_ages_ ≈ 16–17), stronger middle‐wave continuity (Waves 3–8; *M*_ages_ ≈ 17–20), and later attenuation (Waves 9–11; *M*_ages_ ≈ 21–23). Overall, within‐person continuity in MD and PD was strongest mid‐study and weaker at the beginning and end, though standardized estimates were small.

### 3.5 Cross‐Lagged Component

Cross‐lagged associations beyond growth trajectories and autoregressive stability varied across waves (Table A4 & Figure A2). Higher MD predicted lower subsequent PD from Waves 1–2 (*M*_ages_ ≈ 16–17; std est = −0.07, *p* < 0.01) and 10–11 (*M*_ages_ ≈ 22–23; std est = −0.13, *p* < 0.05), but higher PD from Waves 5–6 and 7–8 (*M*_ages_ of Waves 5–8 ≈ 18–20; std est = 0.06 and 0.08, respectively, *p*s < 0.05). Additionally, higher PD predicted subsequent increases in MD from Waves 6–7, 7–8, and 8–9 (*M*_ages_ of Waves 6–9 ≈ 19–21; std est range = 0.05–0.07, *p*s < 0.05). Remaining cross‐lagged paths were not significant. Overall, MD‐PD cross‐lagged effects clustered at specific transitions rather than operating uniformly across waves. However, given the risk for Type I error and small standardized estimates, individual coefficients should be interpreted cautiously, with greater focus on broader patterns.

## 4 Discussion

This study examined the MD‐PD interplay across adolescence and early adulthood in justice‐involved youth to clarify how moral cognition and peer contexts co‐develop. As hypothesized, MD and PD showed evidence of developmental coupling across adolescence and early adulthood. Although both declined on average, their changes were coordinated in timing and pace, with cross‐lagged associations emerging at specific assessment transitions rather than uniformly across waves. In terms of magnitude, the strongest evidence for MD‐PD coupling emerged in the long‐term trajectory associations, whereas autoregressive and cross‐lagged effects were smaller and reflected modest wave‐specific deviations around those broader trends. Directionally, the MD‐>PD paths aligned with selection into, and maintenance of, delinquent contexts, whereas the PD‐>MD paths were consistent with peer socialization of disengaged moral reasoning. Overall, these findings suggest that MD and PD are developmentally linked, particularly through coordinated long‐term trajectories, with more tentative evidence for wave‐specific directional associations.

### 4.1 Latent Growth Curve Component

Consistent with developmental theories, both MD and PD declined from adolescence to early adulthood with significant deceleration, reflecting increasing self‐regulation, consolidating moral standards, and shifting peer contexts as youth transition into adult roles. Although mean scores fell below the scale midpoints, this should not be interpreted as evidence of minimal risk. For MD, scores between disagreement and neutrality may reflect weaker rejection of attitudes that justify, minimize, or reframe harm. For PD, below‐midpoint scores may still capture exposure to delinquent behavior among some friends across multiple behaviors, rather than consistently high exposure across the peer network. Thus, both constructs remained meaningful indicators of individual differences in risk within this justice‐involved sample. Furthermore, these trajectories were strongly interconnected: higher initial MD corresponded to higher starting, slower declining, and earlier leveling PD, while higher initial PD similarly predicted slower reductions and greater deceleration in MD. This coordinated change suggests that moral cognition and peer contexts may co‐develop within a broader developmental system linked to the speed and durability of risk reduction from adolescence to adulthood.

### 4.2 Autoregressive Component

Autoregressive findings showed that short‐term deviations often persisted across the study, indicating that temporary elevations may slow normative declines and sustain risk despite broader improvement. However, this persistence varied across waves: MD and PD carryover was weakest early (*M*_ages_ ≈ 16–17), strongest in middle waves (*M*_ages_ ≈ 17–19), and attenuated as youth entered adulthood (*M*_ages_ ≈ 20–23). These patterns suggest that MD and PD elevations are generally reinforced around late adolescence, when peer networks are relatively stable and sensitivity to peer influence is heightened (Steinberg & Monahan 2007). In younger adolescents, short‐term increases may theoretically reflect situational experimentation with peer affiliation and moral boundaries, producing greater variability and weaker persistence. Then, as youth transition to adulthood, shifting social roles and peer access (e.g., coworkers replacing friends) may reduce reinforcement opportunities and disrupt MD‐PD continuity. Finally, the brief MD reversal at the final transition may indicate a regression to the mean as levels stabilize or end‐of‐study instability (i.e., noise).

### 4.3 Cross‐Lagged Component

Consistent with the unconstrained model's superior fit, the cross‐lag paths varied across waves. At the earliest transition (*M*_ages_ ≈ 16–17), higher MD predicted decreases in PD, which may reflect contextual factors specific to our sample. Specifically, since enrollment followed a serious offense, justice‐system responses such as placement, supervision, or increased monitoring may have restricted youths' opportunities for delinquent peer exposure. In this context, elevated baseline MD may reflect post hoc rationalizations rather than active selection into delinquent peer contexts. Alternatively, peer networks may still be forming in early adolescence, limiting MD's role in selecting into delinquent contexts. In contrast, during the middle waves (*M*_ages_ ≈ 18–20), higher MD predicted increases in PD, consistent with stable peer networks, heightened sensitivity to peer norms, and greater negotiation of moral reasoning within groups (Bandura 1991; Dishion et al. 1996; Steinberg & Monahan 2007). Under these conditions, MD may facilitate involvement with delinquent peers by reducing internal constraints on rule‐breaking and increasing tolerance to antisociality. By early adulthood (*M*_ages_ ≈ 22–23), however, the MD‐>PD association became negative, likely capturing reduced opportunities for delinquent peer involvement as networks destabilize and adult roles expand. Here, elevated MD may represent lingering justifications that persist as youth disengage from delinquent contexts, producing a short‐term negative association. Alternatively, given the deceleration in both trajectories and the small magnitude of this effect, this association may reflect end‐of‐study instability (i.e., noise) rather than a distinct process.

Similarly, PD‐>MD effects followed a complementary developmental pattern. Higher PD predicted subsequent increases in MD during the transition to adulthood (M_ages_ ≈ 18–21), suggesting that sustained exposure to delinquent peers may shape moral reasoning through antisocial socialization, normalization of antisocial norms, and modeling of rule‐breaking justifications. Outside of this window, PD‐>MD associations were nonsignificant, indicating that peer influence on moral cognition is strongest when peer norms are salient and moral standards flexible. Taken together, the directional patterns were selective rather than uniformly reciprocal: MD‐>PD effects appeared at selected early, middle, and later transitions, whereas PD→MD effects clustered from late adolescence into early adulthood. These asymmetrical patterns suggest that disengaged moral reasoning and delinquent peer affiliation may be most closely linked during the middle‐to‐later waves, with the direction and timing of associations varying across transitions. At earlier and later stages, when peer networks may be less consolidated or developmentally reorganizing, these associations were weaker, reversed, or nonsignificant.

### 4.4 Implications

This study positions MD and PD as developmentally linked components of a broader socio‐cognitive risk system, while highlighting the importance of identifying when these processes are coupled rather than assuming they operate as independent, uniform predictors over time. Accordingly, integrated approaches may be especially important and worthwhile investigating during late adolescence and the transition to early adulthood, when MD and PD showed the clearest evidence of coupling in this sample. Future intervention research should test whether combining cognitive‐behavioral programs that strengthen moral reasoning and accountability (e.g., EQUIP; Moral Reconation Therapy; Ferguson & Wormith 2013; Gibbs et al. 1995) with systems‐based approaches that reduce delinquent peer exposure and promote prosocial affiliations (e.g., Multisystemic Therapy [MST]; Henggeler et al. 2009) is useful during this developmental period. Notably, these components may need adaptation for older adolescents and emerging adults (ages 17–26), for whom increasing autonomy, reduced family involvement, shifting social relationships, and changing legal‐system contexts may alter both moral reasoning and peer influence. For example, MST for emerging adults (MST‐EA) retains core MST principles but shifts the primary change agent from parents to the emerging adult while targeting the peer, family, and community systems that maintain risk (Sheidow et al. 2016).

Furthermore, these findings have implications for juvenile justice policy and practice. Probation conditions, placement decisions, and program structures shape youths' social environments and may influence short‐term MD‐PD fluctuations that consolidate into longer‐term risk. Policies and diversion programs that reduce delinquent peer reinforcement while addressing disengaged moral reasoning may help disrupt these self‐reinforcing dynamics and warrant further study. More broadly, institutional decisions in juvenile justice settings may carry unintended developmental consequences that should be considered. However, these implications should be interpreted in light of the study's methodological strengths and limitations.

### 4.5 Strengths and Limitations

A key strength of the present study is its use of 11 waves of longitudinal data spanning adolescence into early adulthood, a critical developmental period for desistance from offending when moral reasoning and peer ecologies undergo substantial reorganization. This sample was also theoretically informative because it included youth with elevated exposure to PD and MD while preserving meaningful variation in their initial levels and developmental trajectories. Moreover, the ALT framework provided a stronger test of transactional processes by separating long‐term change from short‐term within‐person carryover and directional coupling. Finally, measuring PD as behaviors rather than norms improved ecological validity by indexing the peer behaviors most directly implicated in modeling and reinforcement. However, reports of peers' delinquent behavior may also reflect youths' perceptions and broader social projection rather than objective exposure alone. Youth higher in MD may overestimate or selectively perceive delinquency among peers in ways that justify their own behavior, potentially inflating MD‐PD associations. Future studies would benefit from multi‐informant, sociometric, or network‐based assessments that distinguish actual peer behavior from perceived peer norms and projection.

Additional limitations include that the sample was composed of justice‐involved youth from two metropolitan areas, which limits generalizability to individuals from lower levels of system involvement (e.g., community samples), other geographic or policy contexts, and different demographic compositions. The sample was also predominantly male, precluding reliable tests of whether MD‐PD trajectories differed by sex. This is important because peer influence, moral cognition, and pathways to antisocial behavior may operate differently for females, who may be especially affected by relational dynamics and distinct socialization processes (Gini et al. 2014; Moffitt et al. 2001; Rose & Rudolph 2006). Future studies with larger and more gender‐diverse samples should examine whether MD‐PD coupling differs in timing, direction, or strength across sex and gender. Along these lines, because participants were already justice‐involved at baseline, these findings cannot speak to MD‐PD dynamics before serious system contact or the onset of antisocial behavior. Baseline MD and PD may already reflect prior peer selection, socialization, offending, justice‐system responses, or post‐offense rationalizations. Thus, findings are best interpreted as MD‐PD co‐development after justice involvement, rather than the initial emergence of these processes or their role in antisocial onset.

Next, while we attempted to align data by age, these models did not converge, likely reflecting the complexity of estimating an 11‐wave bivariate ALT model with quadratic growth, autoregressive paths, cross‐lagged paths, and within‐wave residual associations. Thus, developmental timing is indexed by assessment wave rather than exact age, which introduced age heterogeneity within waves and limits precision in identifying narrower age‐specific windows of coupling. The unconstrained model also included numerous wave‐specific autoregressive and cross‐lagged paths, increasing the possibility of Type I error. As such, individual coefficients should be interpreted cautiously, with greater emphasis placed on broader developmental patterning than isolated significant effects or precise age‐specific windows. Another related longitudinal measurement issue is that, although Decrop and colleagues (2026) found evidence of longitudinal measurement invariance (LMI) for the MD scale in this sample, LMI of the PD scale was not tested in this study. Accordingly, a change in PD scores may partly reflect shifts in measurement properties over time.

Furthermore, all constructs were assessed via self‐report, raising the possibility of shared method variance and reporting biases for socially sensitive behaviors. Moreover, the study did not examine downstream externalizing outcomes, limiting conclusions about how MD‐PD dynamics translate into behaviors. Additionally, although the ALT framework strengthens temporal inference by separating long‐term trajectories from time‐specific deviations, the observational nature of the data precludes causal conclusions. Relatedly, our models did not include covariates, which allowed us to characterize unadjusted MD‐PD coupling but not covariate‐adjusted prediction. As a result, observed associations may partly reflect shared predictors or time‐varying influences (e.g., psychopathy, parental involvement, peer relationship quality, offense severity, residential placement, supervision intensity).

### 4.6 Future Directions

In addition to addressing these limitations, future research should investigate mechanism‐level specificity in MD‐PD processes, as disengagement strategies (e.g., dehumanization) and forms of PD (e.g., violence, substance use) may differentially shape peer selection and socialization. Studies should also test justice‐system context as a moderator, examining whether changes in supervision, placement, and reentry alter the timing or strength of MD‐PD coupling. Furthermore, researchers should link MD‐PD coupling to varied downstream outcomes such as offending desistance, sobriety, educational and vocational attainment, and prosocial role transitions. Additionally, rather than focusing on overall levels, researchers should test specific signatures (e.g., stronger late adolescence reciprocity, sustained within‐person carryover) to differentiate at‐risk youth. Finally, studies should examine the role of peer contexts in processes of moral re‐engagement where youth abandon disengaged justifications and re‐internalize moral standards.

## 5 Conclusion

MD and PD were developmentally linked across adolescence and early adulthood. Although both declined on average, their trajectories were strongly coupled, such that early elevations in either predicted more persistent elevations and synchronized slowing in the other. Within‐person deviations also showed evidence of short‐term persistence, with small cross‐lagged associations emerging at specific transitions. Overall, these findings suggest that moral cognition and peer contexts co‐develop into adulthood, with the strongest evidence emerging from coordinated long‐term trajectories and more limited evidence from within‐person directional associations. This highlights the potential value of considering both processes, and their timing, as components of a broader socio‐cognitive system that may contribute to cascading risk during the transition to adulthood.

## Author Contributions

**Romain Decrop:** investigation, writing – original draft, methodology, writing – review and editing, formal analysis, conceptualization. **Michael McCart:** supervision, writing – review and editing.

## Funding

The authors have nothing to report.

## Ethics Statement

The original study was approved by the Institutional Review Boards (IRB) at the universities of principal investigators, while this secondary data analysis of de‐identified, publicly available data did not require Bowling Green State University's IRB approval since the study did not meet the definition of human subject research under the purview of the IRB. Ethics approval statement is not applicable.

## Consent

Parental and/or participant consent was obtained when data was originally collected. This consent included permission for researchers to later conduct secondary data analyses like the current study. Information on the study procedures and measures can be found in Schubert et al. 2004 and/or at pathwaysstudy.pitt.edu.

## Conflicts of Interest

The authors declare no conflicts of interest.

## Supporting information


**Table S1:** Pearson Correlation Coefficients of Moral Disengagement and Peer Delinquency. **Table S2:** Items in the Mechanisms of Moral Disengagement Scale. **Table S3:** Items in the Peer Delinquency Scale. **Table S3:** Fit Statistics of Unconditional Growth Trajectories Across Eleven Waves. **Table S4:** Autoregressive Latent Trajectory Model Comparisons.

## Data Availability

The data that support the findings of this study are openly available in ICPSR at https://www.icpsr.umich.edu/web/NAHDAP/studies/29961, reference number 29961.

## Appendix

**Table A1:** Descriptive and Reliability Information Across Waves.

Months from baseline	0	6	12	18	24	30	36	48	60	72	84
Wave	1	2	3	4	5	6	7	8	9	10	11
Mean age	16.04	16.55	17.05	17.52	18.02	18.49	19.01	20.03	21.02	22.03	23.03
Moral Disengagement (Range = 1–3)											
N	1351	1261	1260	1228	1228	1231	1232	1214	1204	1177	1131
Mean	1.62	1.57	1.53	1.50	1.49	1.47	1.45	1.43	1.41	1.38	1.37
SD	0.35	0.36	0.36	0.37	0.36	0.36	0.35	0.35	0.34	0.33	0.34
α of 32 items	0.88	0.90	0.91	0.92	0.92	0.92	0.92	0.92	0.92	0.92	0.93
CWC	—	0.56	0.61	0.64	0.64	0.67	0.66	0.59	0.57	0.57	0.59
Peer Delinquency (Range = 1–5)											
N	1316	1221	1244	1205	1215	1204	1197	1202	1192	1162	1115
Mean	2.32	1.96	1.83	1.78	1.72	1.65	1.61	1.71	1.69	1.67	1.60
SD	0.93	0.87	0.83	0.81	0.80	0.75	0.73	0.78	0.76	0.74	0.70
α of 12 items	0.92	0.89	0.89	0.89	0.91	0.90	0.88	0.88	0.89	0.88	0.87
CWC	—	0.51	0.45	0.42	0.50	0.45	0.47	0.45	0.45	0.47	0.39

*Note:* SD = Standard deviation, α = Cronbach alpha coefficient, CWC = Consecutive wave correlation coefficient.

**Table A2:** Estimates of Unconditional Quadratic Growth Trajectories Across Eleven Waves.

	Growth Factor	Estimate	SE	Variance	SE
Moral Disengagement	Intercept	1.60**	0.01	0.08**	0.01
(*N* = 1354)	Linear	−0.31**	0.02	0.28**	0.03
	Quadratic	0.11**	0.02	0.11**	0.02
Peer Delinquency	Intercept	2.13**	0.02	0.42**	0.03
(*N* = 1351)	Linear	−1.14**	0.06	1.75**	0.25
	Quadratic	0.61**	0.04	0.59**	0.11

*Note:* ***p* < 0.001; SE = Standard error.

**Table A3:** Unconstrained Model—Latent Growth Factor Associations.

	1	2	3	4	5	6
	Std Est (SE)	Std Est (SE)	Std Est (SE)	Std Est (SE)	Std Est (SE)	Std Est (SE)
1. MD Intercept	—					
2. MD Slope	−0.63 (0.06)	—				
3. MD Quadratic	0.52 (0.08)	−0.87 (0.03)	—			
4. PD Intercept	0.60 (0.04)	−0.39 (0.08)	0.36 (0.09)	—		
5. PD Slope	−0.54 (0.07)	0.49 (0.10)	−0.43 (0.11)	−0.74 (0.04)	—	
6. PD Quadratic	0.53 (0.08)	−0.47 (0.11)	0.48 (0.13)	0.66 (0.06)	−0.96 (0.01)	—

*Note:* All *p* < 0.001, *N* = 1354, Std Est = Standardized estimates, SE = Standard errors.

**Table A4:** Unconstrained Model—Autoregressive and Cross‐Lagged Components.

Std Est (SE)		Std Est (SE)	
MD to MD Paths		MD to PD Paths	
MD1 ‐ > MD2	0.05* (0.02)	MD1 ‐ > PD2	−0.07** (0.02)
MD2 ‐ > MD3	0.11** (0.02)	MD2 ‐ > PD3	−0.02 (0.02)
MD3 ‐ > MD4	0.13** (0.02)	MD3 ‐ > PD4	0.01 (0.02)
MD4 ‐ > MD5	0.17** (0.02)	MD4 ‐ > PD5	−0.01 (0.03)
MD5 ‐ > MD6	0.19** (0.03)	MD5 ‐ > PD6	0.06* (0.03)
MD6 ‐ > MD7	0.18** (0.03)	MD6 ‐ > PD7	0.04 (0.03)
MD7 ‐ > MD8	0.16** (0.03)	MD7 ‐ > PD8	0.08** (0.03)
MD8 ‐ > MD9	0.07** (0.03)	MD8 ‐ > PD9	0.06 (0.03)
MD9 ‐ > MD10	−0.04 (0.04)	MD9 ‐ > PD10	−0.03 (0.04)
MD10 ‐ > MD11	−0.19** (0.06)	MD10 ‐ > PD11	−0.13* (0.06)
PD to PD Paths		PD to MD Paths	
PD1 ‐ > PD2	0.09* (0.02)	PD1 ‐ > MD2	0.02 (0.03)
PD2 ‐ > PD3	0.06 (0.02)	PD2 ‐ > MD3	−0.01 (0.03)
PD3 ‐ > PD4	0.07* (0.02)	PD3 ‐ > MD4	0.01 (0.03)
PD4 ‐ > PD5	0.17** (0.03)	PD4 ‐ > MD5	0.03 (0.03)
PD5 ‐ > PD6	0.08* (0.03)	PD5 ‐ > MD6	0.03 (0.03)
PD6 ‐ > PD7	0.12** (0.03)	PD6 ‐ > MD7	0.05* (0.03)
PD7 ‐ > PD8	0.12** (0.03)	PD7 ‐ > MD8	0.06* (0.03)
PD8 ‐ > PD9	0.10 (0.03)	PD8 ‐ > MD9	0.07** (0.03)
PD9 ‐ > PD10	0.09 (0.04)	PD9 ‐ > MD10	0.02 (0.03)
PD10 ‐ > PD11	−0.03 (0.06)	PD10 ‐ > MD11	−0.05 (0.05)

*Note: N* = 1354; MD = Moral disengagement, PD = Peer delinquency, ‐> = path.

** *p* < 0.01

* *p* < 0.05.
