# Supplementary material for: “It's Okay, Everyone Else Is Doing It”: Moral Disengagement and Peer Delinquency
Source: J Adolesc. 2026 Jun 15;98(6):2059–69. doi: 10.1002/jad.70203 (PMC13440693; doi:10.1002/jad.70203)
Supplement: Supplementary file 1 [file JAD-98-2059-s001.docx]

| Supplemental Table 1 | | | | | | | | | | | | | | | | | | | | | | |
| --- | --- | --- | --- | --- | --- | --- | --- | --- | --- | --- | --- | --- | --- | --- | --- | --- | --- | --- | --- | --- | --- | --- |
| *Pearson Correlation Coefficients of Moral Disengagement and Peer Delinquency* | | | | | | | | | | | | | | | | | | | | | | |
|  | 1 | 2 | 3 | 4 | 5 | 6 | 7 | 8 | 9 | 10 | 11 | 12 | 13 | 14 | 15 | 16 | 17 | 18 | 19 | 20 | 21 | 22 |
| 1. MD1 | 1.00 |  |  |  |  |  |  |  |  |  |  |  |  |  |  |  |  |  |  |  |  |  |
| 2. MD2 | 0.56 | 1.00 |  |  |  |  |  |  |  |  |  |  |  |  |  |  |  |  |  |  |  |  |
| 3. MD3 | 0.51 | 0.61 | 1.00 |  |  |  |  |  |  |  |  |  |  |  |  |  |  |  |  |  |  |  |
| 4. MD4 | 0.48 | 0.55 | 0.64 | 1.00 |  |  |  |  |  |  |  |  |  |  |  |  |  |  |  |  |  |  |
| 5. MD5 | 0.46 | 0.53 | 0.59 | 0.64 | 1.00 |  |  |  |  |  |  |  |  |  |  |  |  |  |  |  |  |  |
| 6. MD6 | 0.44 | 0.49 | 0.56 | 0.60 | 0.67 | 1.00 |  |  |  |  |  |  |  |  |  |  |  |  |  |  |  |  |
| 7. MD7 | 0.40 | 0.46 | 0.49 | 0.53 | 0.59 | 0.66 | 1.00 |  |  |  |  |  |  |  |  |  |  |  |  |  |  |  |
| 8. MD8 | 0.40 | 0.41 | 0.49 | 0.51 | 0.53 | 0.59 | 0.59 | 1.00 |  |  |  |  |  |  |  |  |  |  |  |  |  |  |
| 9. MD9 | 0.32 | 0.35 | 0.40 | 0.43 | 0.47 | 0.50 | 0.54 | 0.57 | 1.00 |  |  |  |  |  |  |  |  |  |  |  |  |  |
| 10. MD10 | 0.34 | 0.37 | 0.41 | 0.46 | 0.48 | 0.49 | 0.53 | 0.59 | 0.57 | 1.00 |  |  |  |  |  |  |  |  |  |  |  |  |
| 11. MD11 | 0.33 | 0.37 | 0.35 | 0.42 | 0.45 | 0.44 | 0.47 | 0.51 | 0.50 | 0.59 | 1.00 |  |  |  |  |  |  |  |  |  |  |  |
| 12. PD1 | 0.38 | 0.31 | 0.28 | 0.27 | 0.27 | 0.26 | 0.24 | 0.22 | 0.22 | 0.21 | 0.17 | 1.00 |  |  |  |  |  |  |  |  |  |  |
| 13. PD2 | 0.27 | 0.38 | 0.27 | 0.28 | 0.23 | 0.20 | 0.20 | 0.19 | 0.21 | 0.21 | 0.17 | 0.51 | 1.00 |  |  |  |  |  |  |  |  |  |
| 14. PD3 | 0.24 | 0.27 | 0.34 | 0.29 | 0.29 | 0.25 | 0.24 | 0.26 | 0.22 | 0.21 | 0.19 | 0.44 | 0.45 | 1.00 |  |  |  |  |  |  |  |  |
| 15. PD4 | 0.17 | 0.18 | 0.22 | 0.28 | 0.26 | 0.23 | 0.25 | 0.23 | 0.20 | 0.25 | 0.20 | 0.34 | 0.39 | 0.42 | 1.00 |  |  |  |  |  |  |  |
| 16. PD5 | 0.19 | 0.18 | 0.20 | 0.24 | 0.31 | 0.27 | 0.27 | 0.21 | 0.20 | 0.22 | 0.17 | 0.33 | 0.32 | 0.37 | 0.50 | 1.00 |  |  |  |  |  |  |
| 17. PD6 | 0.20 | 0.21 | 0.24 | 0.27 | 0.31 | 0.32 | 0.31 | 0.24 | 0.26 | 0.22 | 0.24 | 0.31 | 0.28 | 0.39 | 0.39 | 0.45 | 1.00 |  |  |  |  |  |
| 18. PD7 | 0.11 | 0.18 | 0.19 | 0.22 | 0.23 | 0.23 | 0.31 | 0.25 | 0.22 | 0.20 | 0.21 | 0.31 | 0.28 | 0.37 | 0.38 | 0.41 | 0.47 | 1.00 |  |  |  |  |
| 19. PD8 | 0.13 | 0.10 | 0.17 | 0.18 | 0.21 | 0.23 | 0.24 | 0.35 | 0.27 | 0.23 | 0.19 | 0.25 | 0.25 | 0.33 | 0.36 | 0.38 | 0.35 | 0.45 | 1.00 |  |  |  |
| 20. PD9 | 0.17 | 0.13 | 0.18 | 0.21 | 0.28 | 0.24 | 0.26 | 0.27 | 0.31 | 0.29 | 0.21 | 0.24 | 0.25 | 0.30 | 0.34 | 0.38 | 0.38 | 0.43 | 0.45 | 1.00 |  |  |
| 21. PD10 | 0.17 | 0.17 | 0.20 | 0.21 | 0.22 | 0.20 | 0.24 | 0.26 | 0.28 | 0.30 | 0.26 | 0.22 | 0.25 | 0.26 | 0.26 | 0.26 | 0.32 | 0.33 | 0.36 | 0.47 | 1.00 |  |
| 22. PD11 | 0.15 | 0.19 | 0.13 | 0.19 | 0.22 | 0.19 | 0.23 | 0.23 | 0.25 | 0.25 | 0.29 | 0.23 | 0.27 | 0.28 | 0.34 | 0.26 | 0.31 | 0.33 | 0.35 | 0.34 | 0.39 | 1.00 |
| *Note*. All p < .01; MD # = Moral disengagement wave number, PD # = Peer delinquency wave number | | | | | | | | | | | | | | | | | | | | | | |

| Supplemental Table 2 | |
| --- | --- |
| *Items in the Mechanisms of Moral Disengagement Scale* | |
| 1 | It is alright to fight to protect your friends. |
| 2 | Slapping and shoving someone is just a way of joking. |
| 3 | Damaging some property is no big deal when you consider that others are beating people up. |
| 4 | A kid in a gang should not be blamed for the trouble the gang causes. |
| 5 | If kids are living under bad conditions they cannot be blamed for behaving aggressively. |
| 6 | It is okay to tell small lies because they don’t really do any harm. |
| 7 | Some people deserve to be treated like animals. |
| 8 | If kids fight and misbehave in school it is their teacher’s fault. |
| 9 | It is alright to beat someone who bad mouths your family. |
| 10 | To hit obnoxious classmates is just giving them “a lesson.” |
| 11 | Stealing some money is not too serious compared to those who steal a lot of money. |
| 12 | A kid who only suggests breaking rules should not be blamed if other kids go ahead and do it. |
| 13 | If kids are not disciplined they should not be blamed for misbehaving. |
| 14 | Children do not mind being teased because it shows interest in them. |
| 15 | It is okay to treat badly somebody who behaved like a “worm.” |
| 16 | If people are careless where they leave their things it is their own fault if they get stolen. |
| 17 | It is alright to fight when your group’s honor is threatened. |
| 18 | Taking someone’s bicycle without their permission is just “borrowing it.” |
| 19 | It is okay to insult a classmate because beating him/her is worse. |
| 20 | If a group decides together to do something harmful it is unfair to blame any kid in the group for it. |
| 21 | Kids cannot be blamed for using bad words when all their friends do it. |
| 22 | Teasing someone does not really hurt them. |
| 23 | Someone who is obnoxious does not deserve to be treated like a human being. |
| 24 | Kids who get mistreated usually do things that deserve it. |
| 25 | It is alright to lie to keep your friends out of trouble. |
| 26 | It is not a bad thing to “get high” once in a while. |
| 27 | Compared to the illegal things people do, taking some things from a store without paying for them is not very serious. |
| 28 | It is unfair to blame a child who had only a small part in the harm caused by a group. |
| 29 | Kids cannot be blamed for misbehaving if their friends pressured them to do it. |
| 30 | Insults among children do not hurt anyone. |
| 31 | Some people have to be treated roughly because they lack feelings that can be hurt. |
| 32 | Children are not at fault for misbehaving if their parents force them too much. |
| *Note*. Scale from Bandura et al., 1996; Response options: 1 “Disagree,” 2 “Neither agree nor disagree,” 3 “Agree” | |

| Supplemental Table 3 | |
| --- | --- |
| *Items in the Peer Delinquency Scale* | |
| 1 | How many of your friends have purposely damaged or destroyed property that did not belong to them? |
| 2 | How many of your friends have hit or threatened to hit someone? |
| 3 | How many of your friends have sold drugs? |
| 4 | How many of your friends have gotten drunk once in a while? |
| 5 | How many of your friends have carried a knife? |
| 6 | How many of your friends have carried a gun? |
| 7 | How many of your friends have owned a gun? |
| 8 | How many of your friends have gotten into a physical fight? |
| 9 | How many of your friends have been hurt in a fight? |
| 10 | How many of your friends have stolen something worth more than $100? |
| 11 | How many of your friends have taken a motor vehicle or stolen a car? |
| 12 | How many of your friends have gone in or tried to go into a building to steal something? |
| *Note*. Adapted version from Rochester Youth Study (Thornberry et al., 1994); Response options: 1 “None of them,” 2 “Very few of them,” 3 “Some of them,” 4 “Most of them,” 5 “All of them” | |

| Supplemental Table 3 | | | | | | | | | |
| --- | --- | --- | --- | --- | --- | --- | --- | --- | --- |
| *Fit Statistics of Unconditional Growth Trajectories Across Eleven Waves* | | | | | | | | | |
|  | *X*^2^ | DF | AIC | BIC | SABIC | CFI | TLI | RMSEA | SRMR |
| Moral Disengagement (N = 1,354) | | | | | | | | | |
| Intercept only | 1288.08** | 65 | 5014.99 | 5077.52 | 5039.41 | 0.715 | 0.758 | 0.118 | 0.164 |
| Linear | 383.08** | 62 | 3734.61 | 3812.77 | 3765.12 | 0.925 | 0.934 | 0.062 | 0.111 |
| Quadratic | 183.56** | 58 | 3461.96 | 3560.97 | 3500.61 | 0.971 | 0.972 | 0.040 | 0.066 |
| Peer Delinquency (N = 1,351) | | | | | | | | | |
| Intercept only | 929.78** | 65 | 29079.59 | 29142.09 | 29103.97 | 0.612 | 0.672 | 0.099 | 0.142 |
| Linear | 588.53** | 62 | 28587.86 | 28665.99 | 28618.34 | 0.764 | 0.791 | 0.079 | 0.101 |
| Quadratic | 311.66** | 58 | 28175.42 | 28274.38 | 28214.03 | 0.886 | 0.892 | 0.057 | 0.055 |
| *Note*. ** p < .001; *X*^2^ = Chi-square value, DF = Degrees of freedom | | | | | | | | | |

| Supplemental Table 4 | | | | | | | | |
| --- | --- | --- | --- | --- | --- | --- | --- | --- |
| *Autoregressive Latent Trajectory Model Comparisons* | | | | | | | | |
| Model | Description | AIC | BIC | SABIC | RMSEA | CFI | TLI | SRMR |
| 1 | Unconstrained | 30461.38 | 30982.47 | 30664.81 | 0.014 | 0.994 | 0.992 | 0.036 |
| 2 | AR-Invariant | 30517.83 | 30945.12 | 30684.64 | 0.019 | 0.988 | 0.986 | 0.040 |
| 3 | AR + CL Invariant | 30737.96 | 31071.45 | 30868.15 | 0.031 | 0.966 | 0.962 | 0.044 |
| 4 | AR + CL Early/Late | 30697.96 | 31041.87 | 30832.22 | 0.029 | 0.970 | 0.966 | 0.044 |
| 5 | AR + CL PD->MD | 30585.45 | 30965.84 | 30733.95 | 0.024 | 0.981 | 0.978 | 0.041 |
| 6 | AR + CL MD->PD | 30698.90 | 31079.29 | 30847.40 | 0.029 | 0.970 | 0.966 | 0.043 |
| *Note*. N = 1354; AR = Autoregressive paths constrained equal across waves, CL = Cross-lagged paths, MD = Moral disengagement, PD = Peer delinquency; Invariant indicates equality constraints imposed across all waves for both PD->MD and MD->PD, Early/Late indicates cross-lagged paths constrained separately for Waves 1–7 and Waves 8–11, PD->MD and MD->PD models constrain cross-lagged paths in a single direction only. | | | | | | | | |
